# Trans-polar drift-pathways of riverine European microplastic

**DOI:** 10.1038/s41598-022-07080-z

**Published:** 2022-03-17

**Authors:** Mats B. O. Huserbråten, Tore Hattermann, Cecilie Broms, Jon Albretsen

**Affiliations:** 1 Department of Oceanography and Climate, Institute of Marine Research, Box 1870, 5817 Bergen, Norway; 2 Norwegian Polar Institute, Tromsø, Norway; 3 Energy and Climate Group, Department of Physics and Technology, The Arctic University – University of Tromsø, Tromsø, Norway

**Keywords:** Physical oceanography, Marine chemistry

## Abstract

High concentrations of microplastic particles are reported across the Arctic Ocean–yet no meaningful point sources, suspension timelines, or accumulation areas have been identified. Here we use Lagrangian particle advection simulations to model the transport of buoyant microplastic from northern European rivers to the high Arctic, and compare model results to the flux of sampled synthetic particles across the main entrance to the Arctic Ocean. We report widespread dispersal along the Eurasian continental shelf, across the North Pole, and back into the Nordic Seas; with accumulation zones over the Nansen basin, the Laptev Sea, and the ocean gyres of the Nordic Seas. The equal distribution of sampled synthetic particles across water masses covering a wide time frame of anthropogenic influence suggests a system in full saturation rather than pronounced injection from European sources, through a complex circulation scheme connecting the entire Arctic Mediterranean. This circulation of microplastic through Arctic ecosystems may have large consequences to natural ecosystem health, highlighting an ever-increasing need for better waste management.

## Introduction

**Figure 1:**
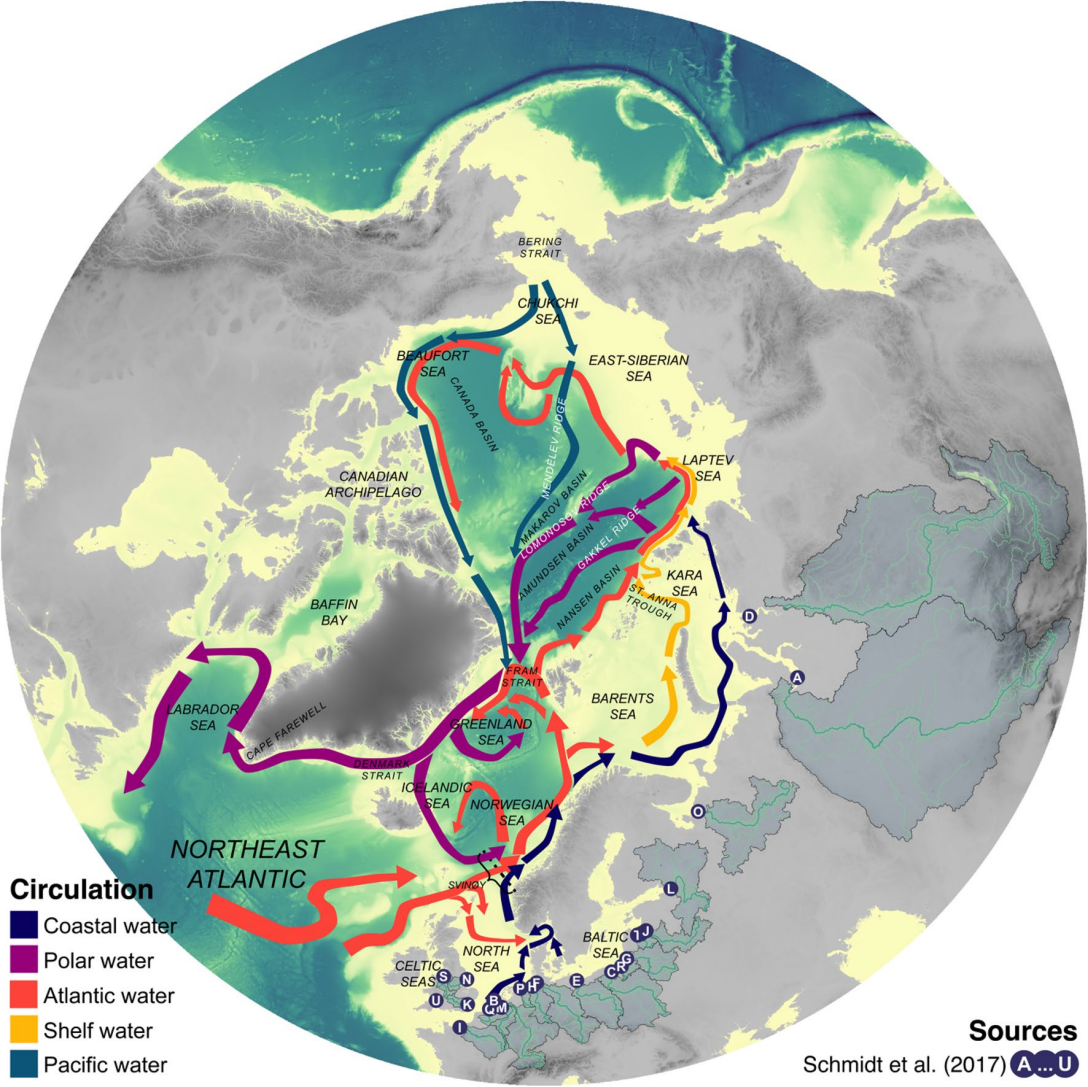
Arctic Mediterranean large-scale circulation and major MP riverine sources. Synoptic overview of Arctic Mediterranean upper layer circulation, based on drift patterns modelled herein and summaries from the literature^20–22^. Letters A-U represent the 21 rivers and drainage basins bordering the Arctic Mediterranean that has an estimated annual microplastic discharge of more than 1 MT^4^, ordered by magnitude of microplastic discharge: A–Ob, B–Rhine, C–Vistula, D–Yenisey, E–Oder, F–Elbe, G–Neman, H–Weser, I–Seine, J–Daugava, K–Thames, L–Neva, M–Meuse, N–Trent, O–Dvina, P–Ems, Q–Pregolya, R–Scheldt, S–Lielupe, T–Mersey, U–Severn. Elevation and bathymetric data is based on ‘the GEBCO grid’ (freely available at https://www.gebco.net), drainage basins and rivers is based on the HydroBASINS and HydroRIVERS data sets^23^, generated using R-packages “raster”^24^ and “sf”^25^.

**Figure 2:**
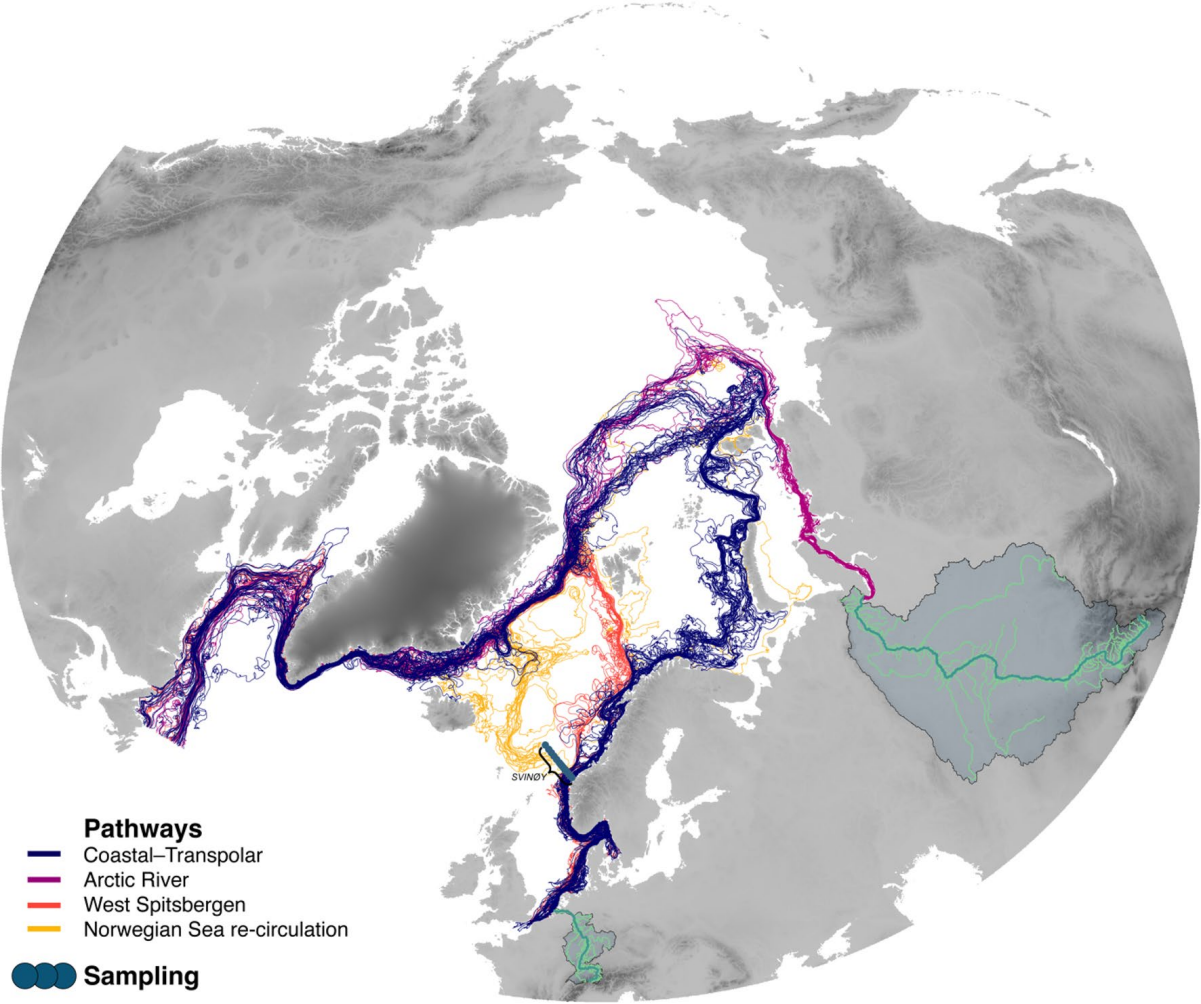
Main drift pathways of European BMP through the Arctic Mediterranean. Here pathways of microplastic particles discharged with the Rhine is used as example (navy blue lines), the largest source of river-borne microplastic in northern Europe (﻿see Schmidt et al.^4^); with alternative pathways along the West-Spitsbergen Current (orange lines); and particles re-circulated in the Nordic Seas, frequently sampled during the Svinøy transect (yellow lines). Also depicted are pathways of plastic particles discharged from Ob river, being the largest estimated point source in the Arctic Ocean (purple lines). Note the small, dark blue dots representing the 17 sampling locations along the standard oceanographic transect “Svinøy”, starting at 62° N off the western coast of Norway (see Figure S1 for detailed map). For map source data, see Fig. 1.

Since the start of plastic production in the 1950s a total of 6300 million metric tonnes (MMT) of plastic waste have been produced globally, and between 8 and 12.7 MMT of this plastic waste reaches the world’s oceans annually^1,2^. A major entry-point of plastic waste to the oceans is by riverine input and, depending on models, between 1.15–2.41 MMT^3^ or 0.41–4.0 MMT are discharged annually, of which more than 50% consists of microplastic (i.e. fraction smaller than 5 mm)^4^. Although 60% of all plastic produced today is positively buoyant in seawater in its original state^5,6^, several open questions remain on mechanisms controlling the duration to which microplastic stays suspended in the water column (i.e. suspension timeline). While it may be deduced that most of the heavy fraction (relative to seawater, e.g. polyvinyl chloride [PVC] and polyethylene terephthalate [PET]) would already sink out of the water column in the river itself or in the immediate vicinity of the river delta^7–9^, the continued suspension of the buoyant microplastic (BMP, e.g. polypropylene [PP], and polyethylene [PE]) may be affected by a multitude of mechanisms^10–17^. However, in reality, an empirically determined suspension timeline of BMP in Arctic waters remains to be quantified. In any case, the continued suspension of BMP in the upper mixed layer may lead to substantial horizontal transport by ocean currents if allowed to enter Earth’s main circulation systems^18^. For example, passive radioactive tracers accidentally released into the coastal waters of northern Europe have been shown to get entrained with the Norwegian Coastal Current (NCC) and disperse widely across the Arctic Ocean within less than a decade^19^. This poleward transport after an initial common path (i.e. the NCC) is made possible by at least three pathways from the Atlantic domain: (1) with the depth integrated mass transport of Atlantic water running counter-clockwise along the Eurasian continental shelf (red arrows, with reference to Fig. 1); (2) with the coastally trapped baroclinic current running along the Eurasian coastline (i.e. along the "Riverine-Coastal domain"^20^, dark blue arrows); or (3) with the mixed shelf water flowing through the Barents, Kara, and Laptev Seas (yellow arrows).

Here we model the long-term dispersal and accumulation of BMP in the upper mixed layer of the Arctic Ocean, discharged from all major, microplastic-polluting drainage basins bordering the Arctic Mediterranean (Arctic Ocean + Nordic Seas)–thus exploring how northern Europe may have acted as a source to the microplastic regularly found in the Arctic Ocean today^26–31^. This was done by applying a 3D, high-resolution, general ocean circulation model of the Arctic Mediterranean, coupled with Lagrangian advection simulations of buoyant particles bound by the upper mixed layer (< 20 m). To explore the dispersive and accumulative potential of BMP flowing into the Arctic Ocean since the arrival of oceanic plastic particles^32^ while maintaining computational feasibility, we cycled our ten-year model archive ad infinitum–assuming a quasi-steady large-scale circulation pattern. Microplastic particles were released from all major rivers across northern Europe and the Arctic that discharge more than 1 MT microplastic annually^3,4^, encompassing drainage basins populated by more than 300 million people, with a total estimated annual discharge of 4000 tonnes of plastic into the regional shelf seas. Our Lagrangian dispersal simulation was subsequently compared to observations of synthetic particles along a transect intersecting the NCC (being the main entrance to the Arctic via oceanic transport), where high concentrations of particles were expected due to the throughflow of most coastal water masses leaving the European shelf seas.

We report a widespread dispersal of BMP along the Eurasian continental shelf, across the North Pole, and eventually back into the Nordic Seas; with several accumulation zones off the continental shelf in the Laptev Sea, over the Nansen Basin, as well as heightened concentrations in the Barents Sea and the oceanic gyres of the Nordic Seas. However, contrary to the expectation of a modal distribution of plastic particles in the NCC, we observed an equal concentration of sampled particles in older and more distal oceanic water masses (at least with reference to timeline of anthropogenic influence). The absence of a distinct gradient in particle concentrations between the proximal NCC and the “older” water masses of the Norwegian Sea interior suggests that microplastic particles has accumulated within the system over decades, through a complex transport and re-circulation scheme connecting the entire Arctic Mediterranean. Moreover, juxtaposing our modelled transport routes onto the contemporary reports of microplastic across the Arctic Ocean, we argue that the only possibility of microplastic ending up at such remote areas is by following the main pathways sketched out here–whereupon a source (i.e. northern Europe) and a corresponding minimum suspension timeline could be identified (at least 4–6 years for European BMP to populate the Eurasian part of Polar basin). These findings are timely contributions to our poor understanding of the distribution microplastic in Arctic waters–but perhaps even more importantly they may heighten our general awareness of the vast dispersal potential of BMP once released into highly advective ocean ecosystems.

## Results

### Trans-polar drift-pathways of northern European riverine BMP

The simulated Lagrangian particles followed two main drift pathways from coastal northern Europe to the high Arctic (the main dispersal pathways are summarized in Fig. 2). The first, and by far the most important pathway was the eastward route, undertaken by roughly 65% of all particles. Although entering the sea at different places along the northern European riverine-coastal domain, most of the particle trajectories merged in the NCC before 62° N, resulting in a coastal (near-land) modal distribution of newly released particles (< 1 year) on the way out of the North Sea. Subsequently particles drifted along the Eurasian continental shelf through the Norwegian, Barents and Kara Seas until ending their eastward drift in the Laptev Sea. After some lingering along the Siberian shelf break the particles where advected into the Arctic Ocean interior across the North Pole, either along the Lomonosov or Gakkel Ridge, upon exiting the Polar basin through the Fram Strait. Overall, few particles visited the Canadian basin of the Arctic Ocean (only about 2.5% of particles ever crossed the Lomonosov Ridge demarking the division between the Eurasian and Canadian parts of the Polar basin), and only 2% of particles exited the Polar basin through the Canadian Archipelago west of Greenland.

The second most frequently followed path was associated with the slope current that bifurcates from the northern Norwegian coast and follows the continental shelf break to the western coast of Spitsbergen, followed by roughly 30% of particles. Most particles following this route re-circulated southward in the Fram Strait, merging with the return flow of the transpolar pathway described above and followed the east Greenland shelf break into the Greenland Sea, where the continued common pathway was along the east Greenland coast, around Cape Farewell, into the Labrador Sea, and further south along the Canadian coastal margin. A fraction of the west-bound particles (20%) re-entered the Nordic Seas subsequent to their visit to the Fram Strait rather than being advected out via the Denmark Strait, as will be integral to the discussions in subsequent paragraphs. In total 19% of particles released from northern Europe never entered the Arctic Ocean and were either deflected into the Norwegian Sea before 66.5° N (roughly demarcating the southern border of the Arctic Ocean), retained in the North Sea close to their river of origin, or were advected into the Northeast Atlantic through the English Channel.

### Accumulation of BMP in the Arctic Ocean

**Figure 3:**
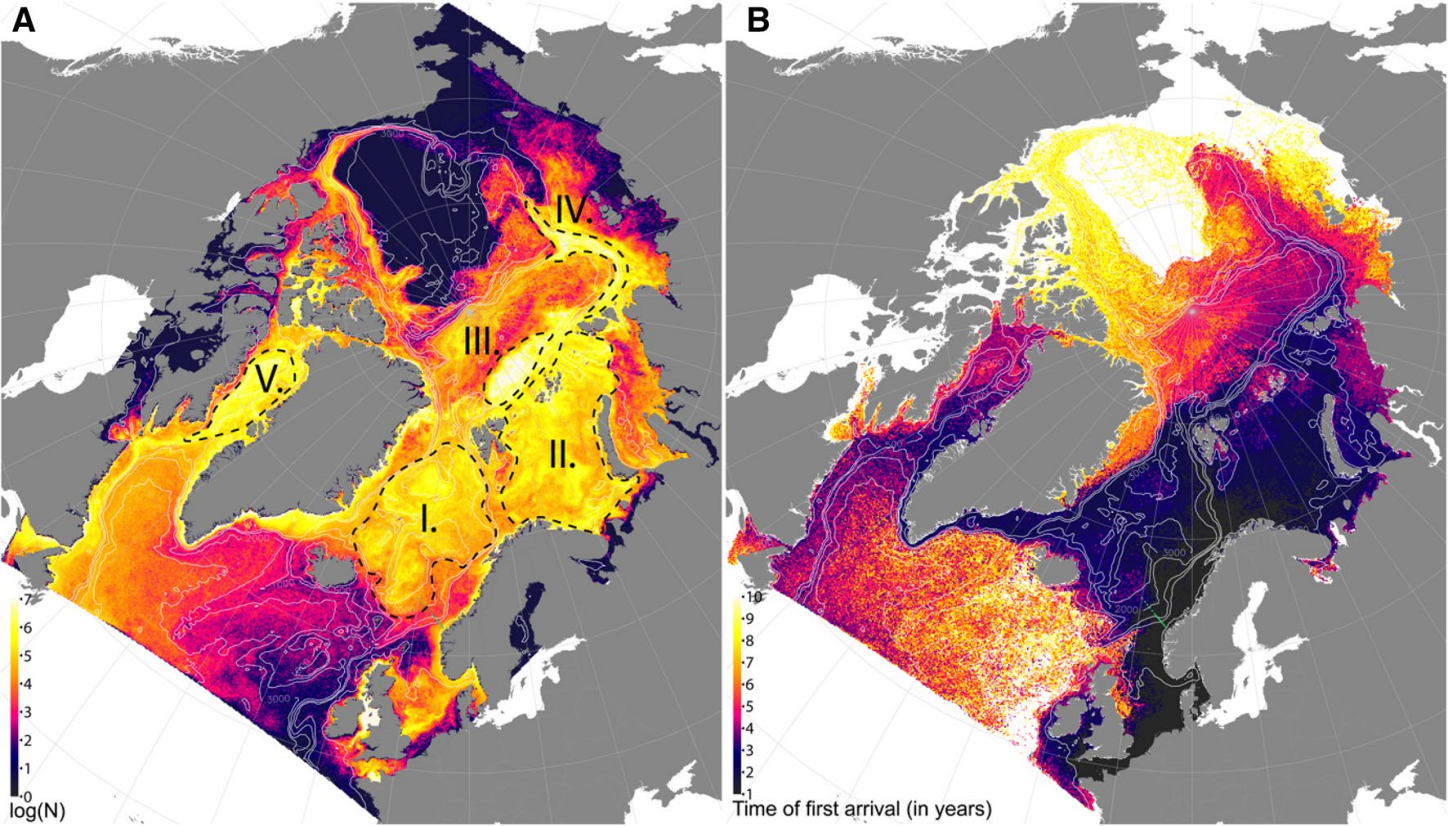
Ocean accumulation zones and estimated time of arrival of European BMP to the high Arctic. (**A**) Integrated abundance of BMP per 4 km × 4 km grid cell over daily concentration fields of the 20th year of simulated advection, plotted on a logarithmic scale. Denoted with roman numerals are the accumulation zones: (I) Nordic Seas, (II) Barents Sea, (III) Nansen Basin, (IV) Laptev Sea shelf edge, and (V) Baffin Bay. Due to the limited ability of the 4 km ocean model grid to resolve near-shore, small scale currents, the apparent accumulation areas near land and in enclosed areas (e.g. the Irish Sea, Franz Joseph Land, eastern coast of Greenland, and Canadian Archipelago) may be artifacts of the model and thus has to be interpreted with caution. Small green dots represent the 17 sampling locations along the Svinøy transect, see also Fig. 2. (**B**) Estimated first time of arrival (rounded to years) of BMP to each 4 × 4 km grid cell of the ocean model, measured as simulated travel time from the North Sea. For map source data, see Fig. 1.

**Figure 4:**
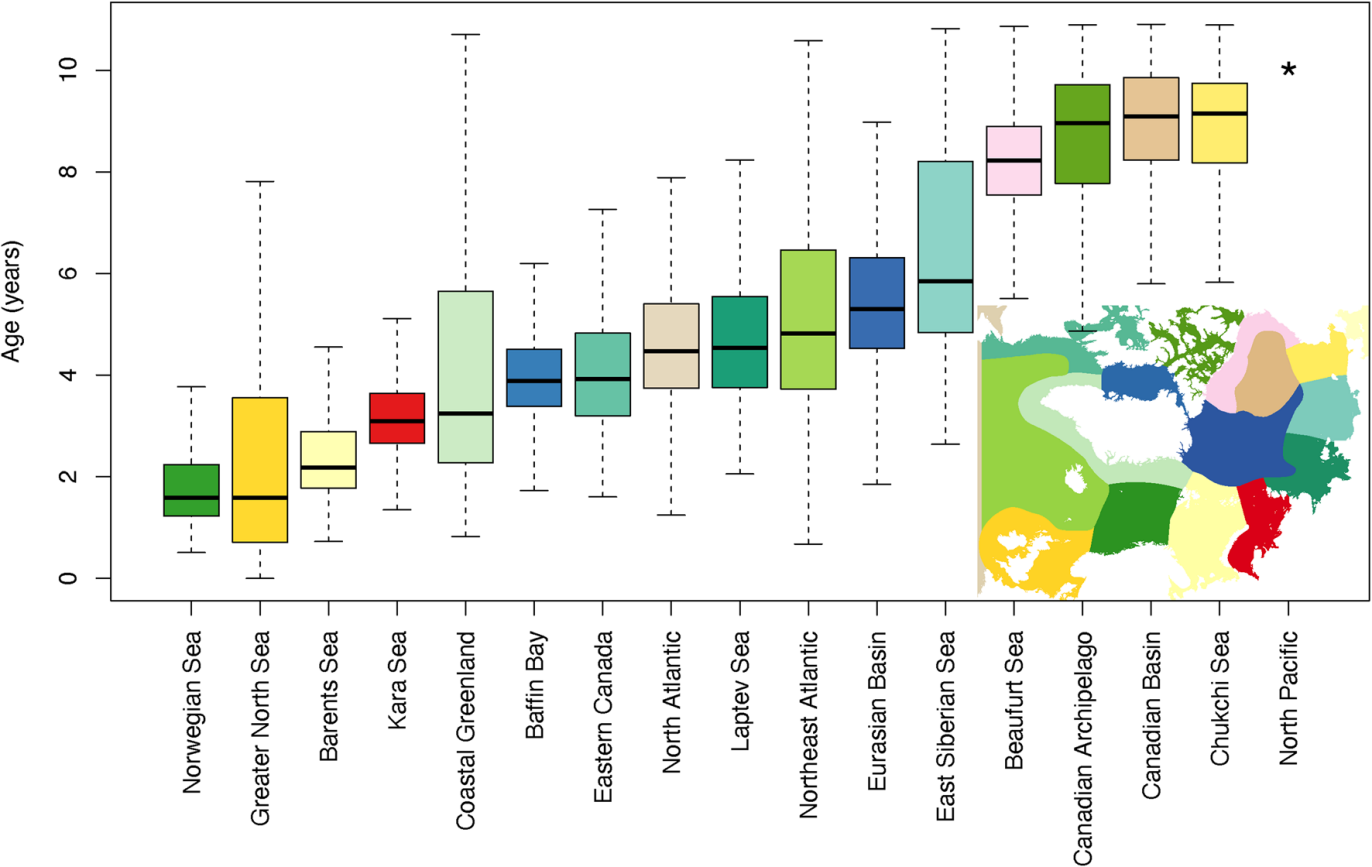
Transport time of European BMP to the regional seas of the Arctic Mediterranean measured as simulated travel time from initial discharge along the North Sea riverine-coastal domain. Here boxes and solid black line represents the 1st, 2nd (median) and 3rd quartile of the distributions, the “whiskers” 1.5 × interquartile range, and open circles outliers. Color of boxes refers to the corresponding regional sea or area which extent is demarcated in small inset. *Note that no particles entered the Bering Sea (North Pacific) in our simulations, thus no minimum age could be estimated.

A clear spatial pattern of aggregation of particles emerged after 20 years of advection, with distinct accumulation zones (defined as areas of elevated particle concentrations) in the Barents Sea; along the continental slope in the Laptev Sea stretching onto the Lomonosov Ridge; over the Nansen Basin; in the ocean gyres of the Greenland, Icelandic, and Norwegian seas; and in Baffin Bay (Fig. 3A). This overall picture seemed stable and did not change much after 30 years of advection (i.e. after ten additional years, see “Methods” section) except a slight decrease in overall concentrations due to advection out of the model domain, suggesting that the model area was fully saturated with particles (except the Chukchi Sea and central parts of the Canadian Polar basin) and an equilibrium in spatial distribution was achieved already before 20 years. First time of arrival of modelled plastic particles from the heavily populated and industrial areas around the North Sea to the regional seas of the Arctic Mediterranean ranged from < 2 years to the Norwegian Sea, between 4 and 6 years to the Eurasian part of the Polar basin, and up to 10 years for any particles to reach the regional shelf seas of the Canadian Arctic (Fig. 3B and see summary statistics of time of first arrival integrated across the individual regional seas in Fig. 4).

**Figure 5:**
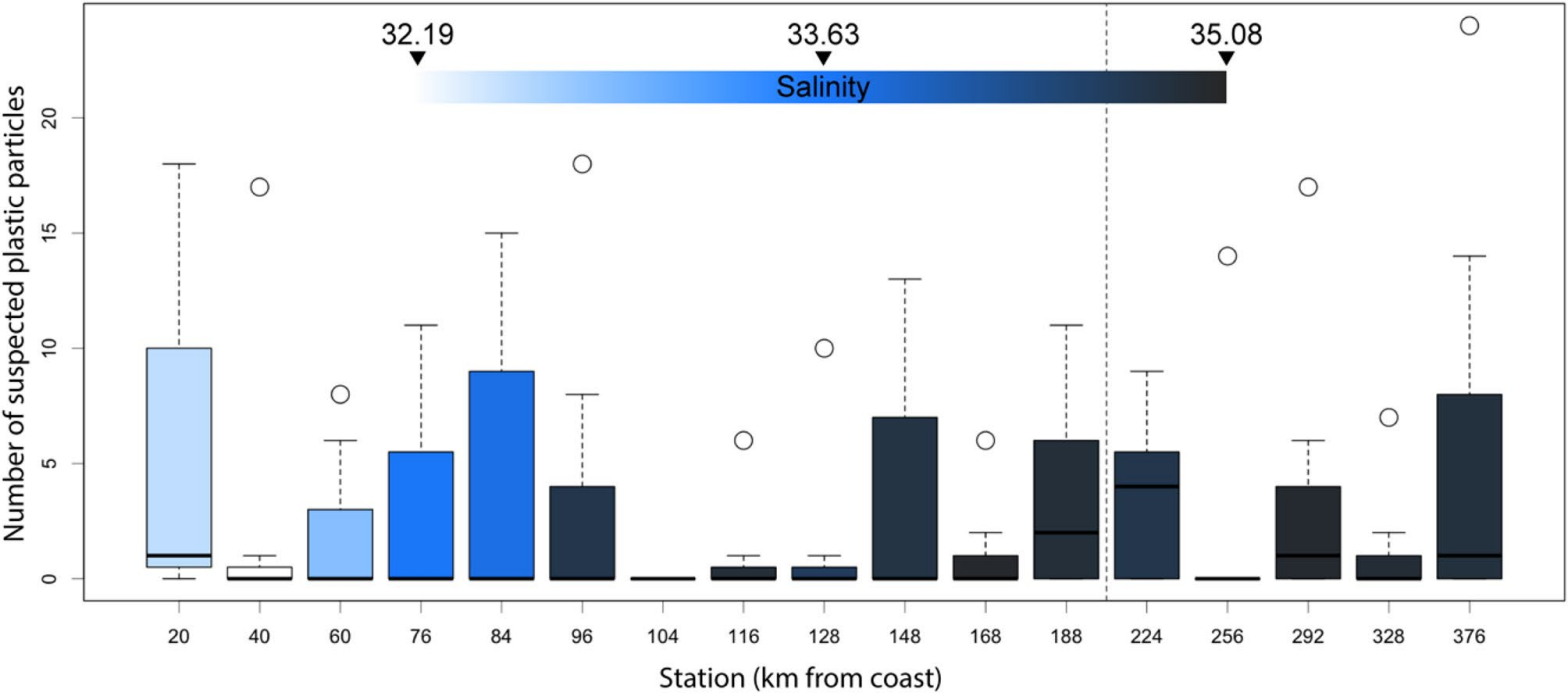
Counts of suspected plastic particles and fibers in vertical plankton hauls along the Svinøy transect, traversing the Norwegian Coastal Current, the Norwegian Atlantic Current, and the south-eastern Norwegian Sea. Here blue hues of colored boxes indicate the average salinity at the fixed station across the seven synoptic transects made, and dashed line after 188 km demarcates the split between the inner and outer segment of the transect as referred to in the text. For interpretation of “box and whisker” plot see Fig. 4 legend.

**Figure 6:**
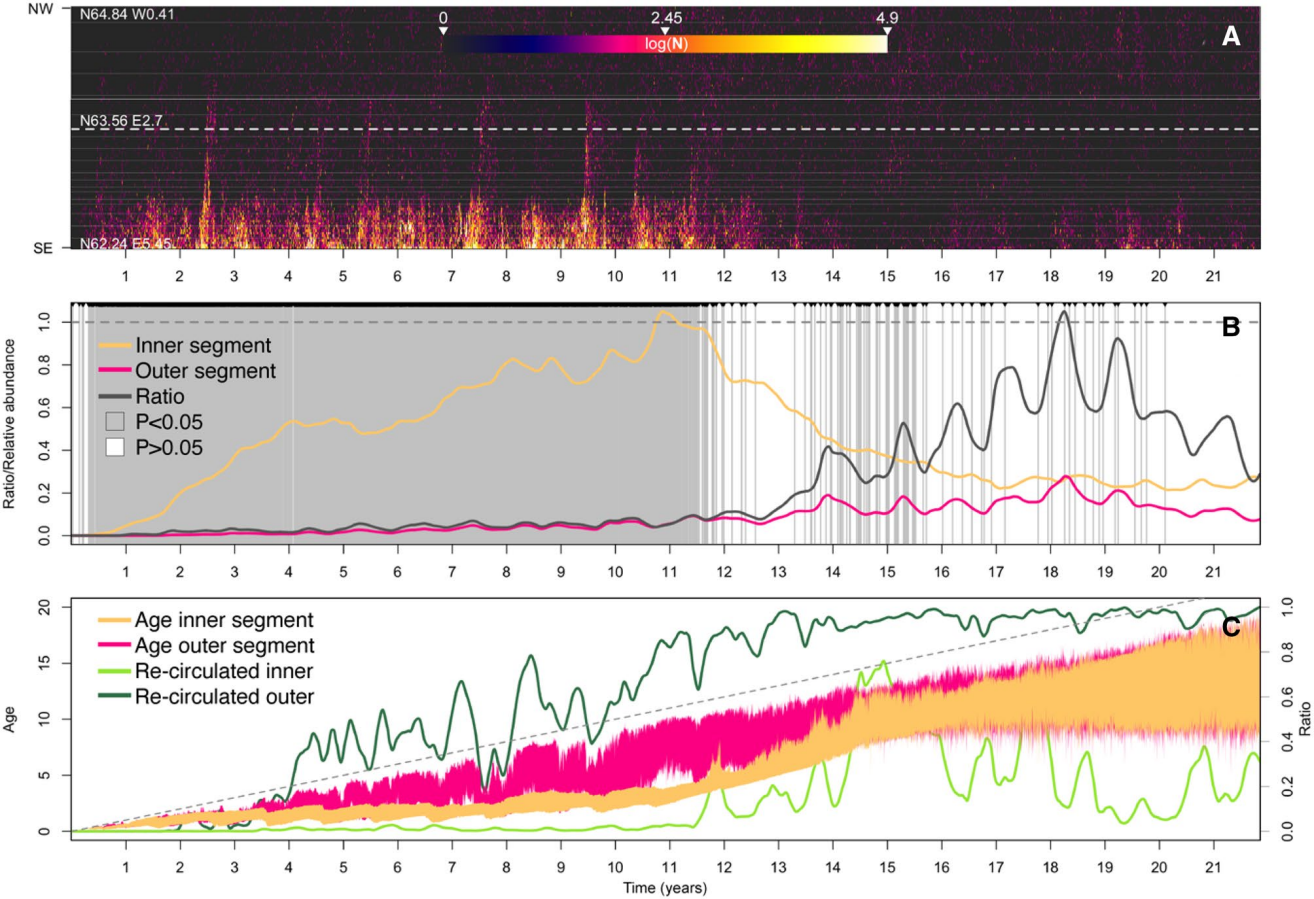
Temporal development of modelled flux of particles through the Svinøy transect. (**A**) Hovmöller diagram representing the modelled flux of BMP through the standard oceanographic transect “Svinøy” during the drift experiment, with thin, horizontal, white lines representing the position of the 17 fixed sampling stations. (**B**) Relative abundance of modelled particles in the inner (yellow line) and outer (pink line) segment of the transect, and ratio between the two segments (dark grey line) as divided by white perforated line in panel (**A**). Also plotted are the results from sequential nb-glmms (one for each day, grey/white vertical lines) replicating the sampling and testing scheme applied to the empirical data. (**C**) Confidence bans (1st to 3rd quartile) of modelled age of particles flowing through in inner (yellow band) and outer segment (pink band) of the Svinøy transect, and proportion of re-circulated particles (i.e. frequency of particles that already had visited the Fram Strait divided by total amount of particles drifting through the transect) in the inner section (light green line) and outer section (dark green line).

Counts of suspected synthetic particles, sheets, and fibers sampled along the standard oceanographic transect “Svinøy”, intersecting the NCC, the Norwegian Atlantic Current (NAC), and southeastern parts of the Norwegian Sea revealed a highly variable, yet flat spatial pattern (Fig. 5). In total, 345 suspected plastic particles were identified in the 121 vertical net hauls performed at 17 fixed stations over seven synoptic transects, yielding an average concentration of 0.06 (0.11 std.dev.) particles per m^3^ of seawater sampled. The distribution of sizes of particles/sheets and fibers was highly skewed towards the smaller sizes, with a median of 1 mm (1st and 3rd quantile 0.7–1.7) for particles/sheets and 3.2 mm (1st and 3rd quantile 2.27–5.1) for fibers (see images of all particles and size distributions in Figure S2). A negative binomial generalized linear mixed model (nb-glmm) with counts of plastic particles in the inner section (sampling the NCC) and outer section (sampling the southeastern boundary of the Norwegian Sea) of the Svinøy transect as co-factors suggested slightly higher concentrations in the Norwegian Sea (yet not significantly different) compared to the NCC (exp(α) = 2.8 vs. exp(α + β_outer_) = 3.6, z = − 0.52, p = 0.59). Thus, the hypothesis of a modal distribution of plastic particles in the NCC, associated with a pronounced inflow from northern Europe into the Arctic Ocean could not be supported by the observed distribution. Instead, the observed distribution of sampled particles across the transect that was more consistent with the modelled particle distribution after a decade of accumulated discharge and circulation from northern European rivers (Fig. 6), when the particles already had the time to saturate/permeate the entire model area after either having re-entered the Nordic Seas via the Polar basin; or had been re-circulated across the Fram Strait.

## Discussion

Combining the high persistence of BMP in the water column with the vast dispersal potential provided by the Arctic Mediterranean large-scale circulation system it is not surprising that plastic particles are found across the entire Arctic Ocean. However, in contrast to the idealized circulation scheme of the Arctic Ocean where the bulk flow enters the Polar basin through the deeper Fram Strait^21,33,34^, the main transport route of the European derived BMP was with the freshwater-influenced water masses of the Eurasian riverine-coastal domain and the mixed shelf water flowing all the way from the greater North Sea area to the Barents, Kara and Laptev Seas of the high Arctic^20,21,35^. This qualitative difference to the expected drift route most likely arose through differences in drift depth (upper mixed layer vs. intermediate depths of bulk flow) as also have been shown by radioactive tracer analysis^19,36,37^. Sensitivities to drift depth probably also influenced the departure taken from the Eurasian shelf with the Trans-Polar Drift when reaching the Laptev Sea, as opposed to a continued drift with the sub-surface boundary current into the Canadian basin. While the exact point of departure the Eurasian freshwater makes from the continental shelf once reaching the Laptev Sea varies by the prevalent wind field, the Eurasian contribution to the Trans-Polar drift usually follows the Gakkel or Lomonosov Ridges^38–41^.

Another vessel for transportation across the North Pole is within the pack ice itself; for example being incorporated into ice in the Laptev Sea and later being re-suspended in the Arctic surface water when the pack ice melts én-route to the Greenland Sea^42–44^. However, although the mechanisms incorporating plastic into the ice have been identified on a conceptual level^10^, dedicated studies on specific uptake parameters are needed for development of proper sub-modules representing realistic plastic-in-ice-drift in general dispersal models. Snow on ice floes has also been shown to contain high numbers of the smallest fraction of microplastic (< 25 µm), most likely deposited from the atmosphere^45^. In any case the vast majority of the ice-associated particles eventually end up in the East Greenland Current (EGC) being the main exit point of both liquid and solid freshwater from the Arctic Ocean, albeit with a delay of up to three years if trapped in the sea ice^35,46^. Across-pole transport of BMP from the North Pacific via the Bering Strait is also likely taking place (although not modelled here); however given the tenfold differential in Pacific vs. Atlantic throughflow of water masses^47–49^, the Atlantic domain sources are most likely the dominant drivers of the observed distribution of BMP in the Arctic Ocean.

The identified drift pathways connected the entire Arctic Mediterranean within a timescale of less than a decade, with at least four well-defined accumulation zones of European derived BMP spread across the study area. The first, yet least obvious accumulation zone based on prior oceanographic understanding was the Laptev Sea shelf break, between Zevernaya Zemlya and the Lomonosov Ridge. Our simulations suggest that at least 65% of all particles released along the northern European coast visits the Laptev Sea during its horseshoe-shaped journey through the Arctic Mediterranean, and most of these pathways entering the Laptev Sea appeared to be confined to the front between the less saline shelf waters and the highly seasonal shelf break current^33^. This is also an area of intense ice production constituting nearly 10% of the yearly produced Eurasian drift ice^43,46,50^. Ice cores created in this Laptev Sea flaw lead contain high concentrations of varnish and antifouling paint from ships that has been accredited to local shipping and fishing activity^42^. However, according to our simulations these particles are not necessarily of local origin as this appears to be the prime staging area for the majority of BMP released into the Eurasian riverine-coastal domain before undertaking their eventual Trans-Polar drift. This notion is corroborated by observations from the Laptev Sea where higher concentrations of microplastic are found in water masses with an Atlantic influence than within the more locally influenced, coastal water masses^30^.

A second, uncharted accumulation zone was situated above the Nansen basin, where microplastic have been found in large quantities in the upper mixing layer^27^, and ice cores formed above the basin have been shown to contain high concentrations (> 10^7^ N m^−3^) of buoyant PE particles^42^. These PE particles may have been transported from the Norwegian Sea by the West Spitsbergen Current^29^, or with the Trans-Polar drift originating in the Laptev Sea, either suspended in the Arctic surface waters or deposited by the pack ice during summer melting^43^.

A third focal area of the plastic particles circulating through Arctic Mediterranean was the aggregated Nordic Seas accumulation zone (i.e. Icelandic, Greenland and Norwegian Seas) encompassing the gyres forming between the incoming Atlantic water masses from the southeast, and the Arctic water masses from the northwest^22^. High concentrations of microplastic have been found in the Greenland Sea^28^, and in the northeast Atlantic^51^. However, contrary to the expectation of a well-defined modal distribution of plastic particles in the NCC, due to its parent water masses originating along the nearby heavily populated and industrialized coastal areas of northern Europe, particles were equally abundant in the more distal, oceanic water masses of the Norwegian Sea. Although the Svinøy transect crosses an area of complex mesoscale dynamics^52,53^, most likely adding some statistical “randomness” of our particle counts along the sampled transect; the convergence of observed and modelled variables more than ten years into our dispersal scenario suggests that the Arctic Mediterranean is currently in an advanced accumulative state with regards to the saturation of BMP, through the complex transport, accumulation, and re-circulation scheme highlighted in the present study.

While it is evident that the plastic particles reported across the Arctic Ocean today may have a rich history with regards to possible sources and pathways taken to get there‚ the qualitative distribution is highly parsimonious to a scenario of historical discharge and accumulation of microplastic originating from the Eurasian riverine-coastal domain. The observed resemblance of the Arctic Mediterranean being in an accumulative state strongly implies that buoyant microplastics and other synthetic particles must remain suspended in Arctic surface waters for a very long time (up to decades). This circulation of extremely durable, floating plastic through the regional Arctic ecosystems may have far reaching consequences, for example by ingestion by invertebrates, fish, birds, and mammals^17,54–56^–inducing a wide array of detrimental physiological responses^57^. Our findings may also heighten the general awareness of the vast dispersal potential of BMP once released into highly advective ocean ecosystems. The present research thus serves as timely arguments for policymakers to regulate key attributes of plastic products (i.e. density and durability) and highlights the importance of an ever increasing focus on better waste management, in turn reducing the potential for dispersal of terrestrial microplastic in marine ecosystems.

## Materials and methods

### Ocean model and particle advection scheme

The hydrodynamic model used to represent the ocean currents in the study area was based on metROMS (https://doi.org/10.5281/zenodo.290667), which couples the state-of-the-art Regional Ocean Modeling System (ROMS, http://myroms.org), a free-surface, hydrostatic, primitive equation ocean general circulation model^58^, with the comprehensive dynamic-thermodynamic sea ice model CICE (https://zenodo.org/record/1205674). metROMS was run with a horizontal resolution of 4 × 4 km in an orthogonal, curvilinear grid covering the entire Arctic Mediterranean over the time period 2007–2017, referred to as the “A4-setup”^53,59^. The output from the A4-setup contained velocity fields in 32 terrain following vertical layers, and a temporal resolution of 24 h. To enable a test of our hypothesis of a perpetual suspension of buoyant microplastic in the Arctic Ocean the A4-archive was cycled in subsequent 10-year periods, as specified further in the next paragraph. The advection of particles in the horizontal plane was modelled by the Runge–Kutta fourth order scheme LADiM^60^, evaluated every hour of the simulation yielding 3986 time steps per 10 year cycle of the A4-archive. To model the generally unknown vertical movement of buoyant microplastic particles over annual time scales, the particles was bound by a random vertical walk limited within the upper 20 m of the water column, moving up to 10 cm up or down every hour. Although this approach is likely to be a gross simplification of the complex interplay between the buoyancy of the particle, mechanical fragmentation, microbial degradation, ingestion by organisms, biofouling, flocculation, ice encapsulation, and turbulence (as reviewed briefly in the introduction)—little to no empirical data is available from the Arctic Ocean to calibrate all the sub-models necessary to assemble a holistic dispersal model based on first principles.

### Particle release scheme and sensitivity analyses

The particle dispersal ensemble was initiated with the number of particles proportionally to the estimated microplastic river discharge estimated by^4^ “model 1”. Major rivers discharging more than 1 t of microplastic into the Arctic Mediterranean include (decreasing microplastic discharge measured in metric tonnes, A-U in Fig. 1): Ob (796t), Rhine (473t), Vistula (317t), Yenisey (118t), Oder (98t), Elbe (77t), Neman (41t), Weser (25t), Seine (14t), Daugava (12t), Thames (12t), Neva (11t), Meuse (11t), Trent (4t), Dvina (3t), Ems (3t), Pregolya (2t), Scheldt (2t), Lielupe (1t), Mersey (1t), and Severn (1t). To identify realistic point sources (i.e. river-mouths) for where particles should be released we used the HydroRIVERS and HydroBASINS data sets of the European and Siberian regions^23^. Do note that the Baltic Sea was only implicitly modelled in the A4-setup by its freshwater discharge into the North Sea, thus the flow of particles released into the Baltic was not explicitly modelled here (i.e. Vistula, Oder, Neman, Daugava, Neva, Pergyola, Lielupe). However, it is well known that the freshwater outflow from the Baltic mixes with the freshwater from the southern North Sea in the Skagerrak^61^. Together these two freshwater sources forms the basis of the NCC, and any downstream path beyond the Skagerrak is indistinguishable. Particles were released from each major river within the model domain, every day over ten years with same number each day, as no temporally varying source function exists in the literature (in total 403.078 particles). After an initial spin-up phase of ten years of daily releases the geographical coordinates of all particles in the model was exported to a new 10-year cycle of the A4-archive, initiated with the same positions that the particles had obtained at the end of the previous cycle. The process of re-sampling the A4-archive was repeated as long as needed for particles to saturate the entire model area, but no new qualitative outcomes were obtained after the end of the third cycle (i.e. after a 30-year simulation). However, do note that no new particles were released after the initial ten year spin-up.

A sensitivity analyses was performed on the importance of initial conditions (i.e. particle release positions and magnitude). First and foremost, an alternative release scenario was run based on modelled daily average freshwater discharged from each river in the European Flood Awareness System hindcast^62^, a specific application of the hydrological model LISFLOOD^63^. The number of release positions was subsequently narrowed down to rivers of drainage basins that had an average discharge higher than 20 m^3^ s^−1^, a total population above 1000, and a catchment area above 1000 km^2^, yielding a total of 230 rivers distributed along the northern European coastline. However, this release scheme did not produce any different outcome with regards to accumulation zones or main pathways due to the highly unified pathways of all freshwater released onto the Eurasian shelf seas. Moreover, to explore if Northeast Atlantic import of plastic particles to the Nordic Seas would qualitatively affect the flux of particles through the Svinøy transect we also ran a separate drift experiment where particles were released across the Rockall Trough. Here the vast majority of particles drifting eastwards followed the coastal water west of the British Isles and into the North Sea with the Fair Isle Current, eventually exiting the North Sea with the NCC, though at a considerable delay relative to the particles discharged into the North Sea proper.

### Field sampling of synthetic particles and statistical inference

A total of seven synoptic sampling transects were made along a straight line between 62.37° N–5.20° E to 64.67° N–0.00° E in the time period May 2017 to August 2018 (see Fig. 2 for general sampling locations, and Figure S1 detailed map). Synoptic transects usually lasted 3–4 days (from first to last station) and were performed (roughly) on a bi-monthly basis. Here the inner segment represented the stations that sampled the NCC (stations 1–12), and the outer segment the stations sampling the outer fringe of the Norwegian Sea gyre (station 13–17). At each station a CTD and a WP2 (zooplankton net) integrated vertical haul was performed, from 200 m to the surface. The WP2 net had an opening of 0.25 m^2^ and a mesh size of 180 μm, sampling on average 50 m^3^ of seawater. All the plankton samples were inspected under the dissection microscope and all particles, sheets, and fibers suspected to be made of plastic or synthetic materials were counted, measured, weighed (after drying), and classified according to morphology and color. Do note that although much of the fibers collected in water samples worldwide are natural fibers (e.g. cellulose) from the textile industry and not necessarily plastic per se^64^; for the purpose of this study all fiber likely follow the same dispersal dynamics, given equal density and shape characteristics. Moreover, to control for possible contamination of samples, a total of three blanks were analyzed. Here sampling equipment was flushed with the on-board hoses instead of sampling the water column, all else treated equal to “normal” samples. A total of four fibers were recovered among the three blanks (three in blank no. 1, and one in no. 2), suggesting a low, yet not insignificant source of fiber contamination during treatment of samples.

To test if the concentration of synthetic particles and fibers from northern Europe transported northwards with the NCC was higher than the concentration of particles in the Norwegian Sea (i.e. if the counts of particles in the inner and outer segment of the Svinøy transect was different), a negative binomial, generalized linear mixed model (nb-glmm) was fitted to the counts of particles, with inner and outer segment as a two-level factor and the seven synoptic transects as a random intercept. In the model selection phase we first tried a generalized linear model (GLM) with a Poisson distribution of the residuals. However, the Poisson count-model showed a high degree of overdispersion, meaning that the variance was higher than the mean as expected under the Poisson distribution^65^. To account for the variance being higher than expected of a count, a negative binomial GLM (nb-GLM) was fitted to the data, which decreased the AIC from 1032 to 444 compared to the plain count model. Furthermore, since single measurements along the synoptic transect was not independent in a strict sense, the transect number (1–7) was added as a random intercept to the nb-GLM. The addition of the random effect in the final nb-glmm lowered the AIC to 438.

## Supplementary Information

Supplementary Figures.
