# Supplementary material for: Trans-polar drift-pathways of riverine European microplastic
Source: Sci Rep. 2022 Mar 17;12:3016. doi: 10.1038/s41598-022-07080-z (PMC8931020; doi:10.1038/s41598-022-07080-z)
Supplement: Supplementary file 1 — Supplementary Figures. [file 41598_2022_7080_MOESM1_ESM.docx]

# Supplemental materials

**
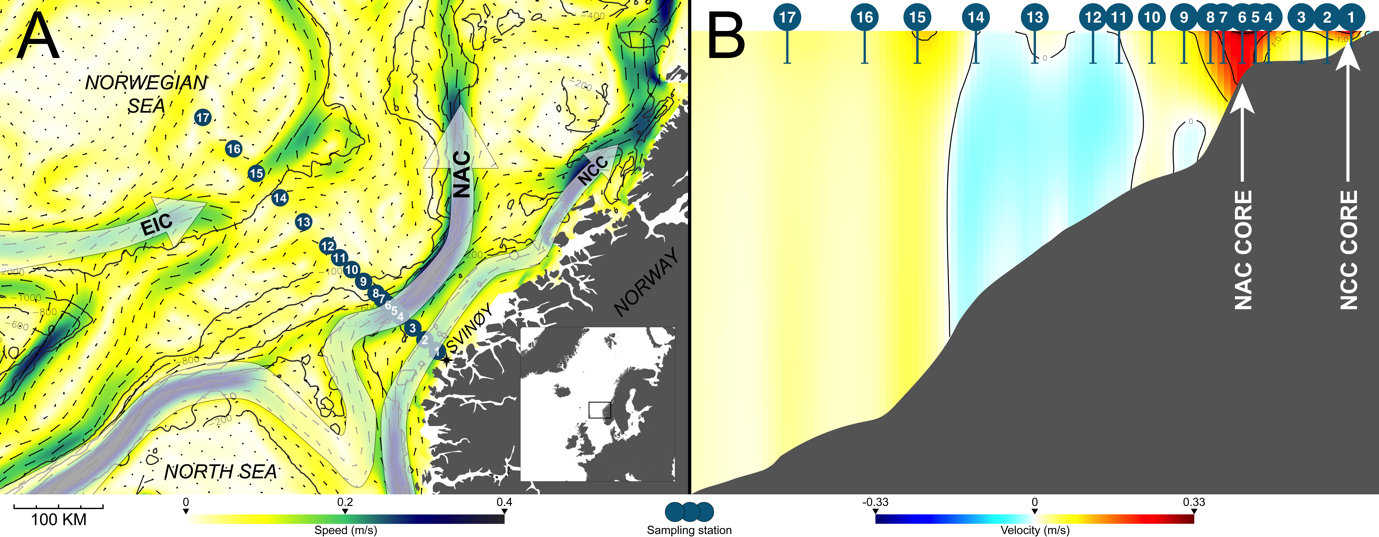
Figure S1. Detailed map of MP sampling stations.** (**A**) Map of 17 sampling locations used in this study along standard hydrographic transect Svinøy, with modelled current speed averaged over upper 20 m and ocean currents referred to in text: the Norwegian Coastal Current (NCC), Norwegian Atlantic Current (NAC) and East Icelandic Current (EIC). (**B**) Vertical current structure along transect Svinøy relative to sampling depth of the integrated vertical WP2 hauls at 200 m. Here positive velocity (warm, red gradient) represents currents flowing towards north-east (normal to the transect), while negative velocity (cold, blue gradient) means currents flowing towards south-west. For map source data, see Figure 1


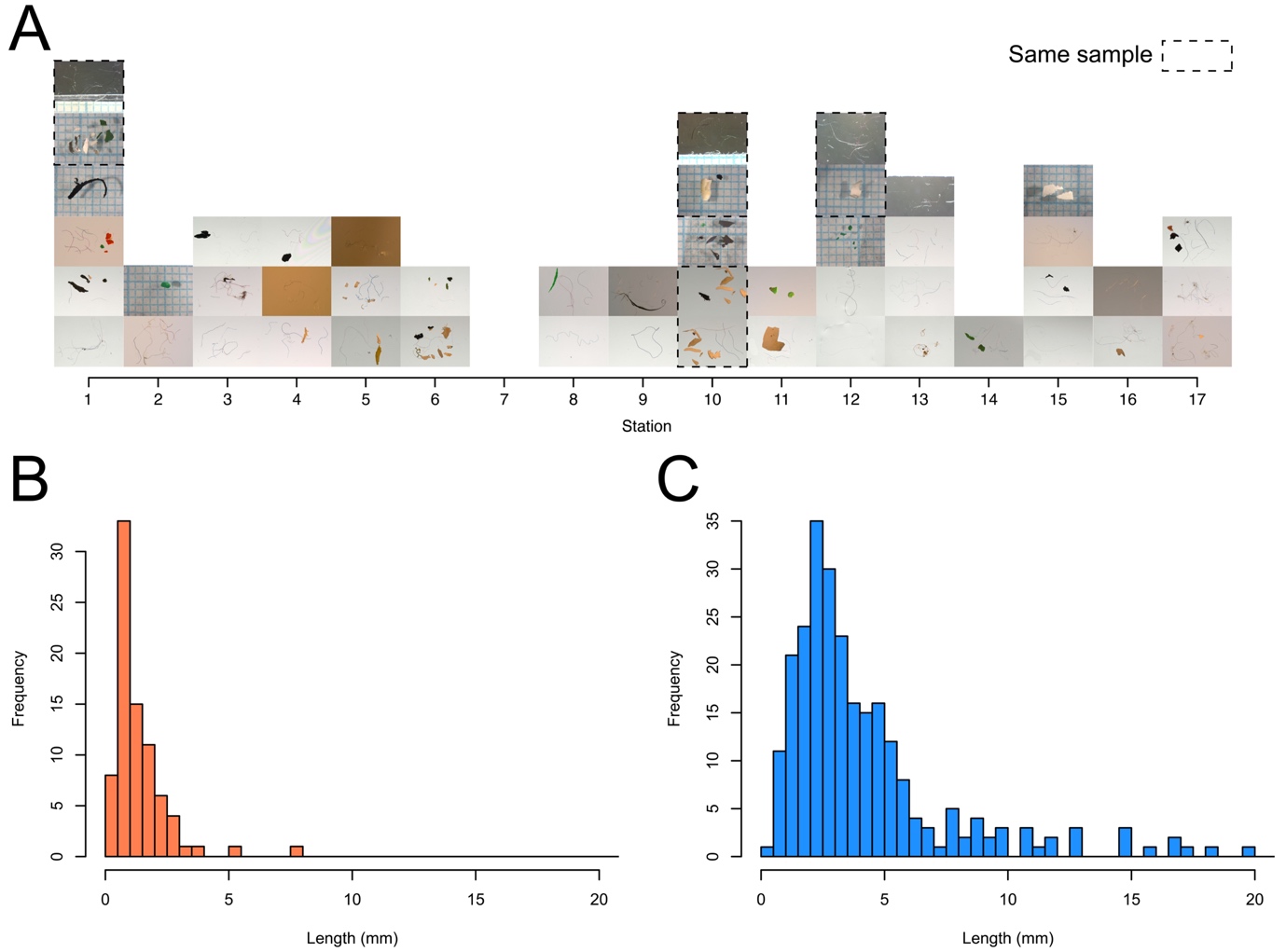


**Figure S2. Images of suspected microplastic particles and distribution of particle sizes.** (**A**) High resolution images of suspected microplastic particles sorted from each zooplankton sample, and organized according to position along the 17 stations constituting the fixed hydrographic transect “Svinøy” (see Figure 2 for geographic position of transect, and Figure 5 for per station counts of particles). (**B**, **C**) Size distribution of identified suspected microplastic particles, divided into general categories: (B) fragments and sheets; and (C) fibers.
